# Probabilistic identification of saccharide moieties in biomolecules and their protein complexes

**DOI:** 10.1038/s41597-020-0547-y

**Published:** 2020-07-03

**Authors:** Hesam Dashti, William M. Westler, Jonathan R. Wedell, Olga V. Demler, Hamid R. Eghbalnia, John L. Markley, Samia Mora

**Affiliations:** 1 Center for Lipid Metabolomics, Division of Preventive Medicine, Department of Medicine, Brigham and Women’s Hospital and Harvard Medical School, Boston, 02215 Massachusetts USA; 2 Department of Biochemistry, National Magnetic Resonance Facility at Madison and BioMagResBank, University of Wisconsin Madison, Madison, 53706 Wisconsin USA; 3 Cardiovascular Division, Department of Medicine, Brigham and Women’s Hospital and Harvard Medical School, Boston, 02215 Massachusetts USA

**Keywords:** Carbohydrates, Software

## Abstract

The chemical composition of saccharide complexes underlies their biomedical activities as biomarkers for cardiometabolic disease, various types of cancer, and other conditions. However, because these molecules may undergo major structural modifications, distinguishing between compounds of saccharide and non-saccharide origin becomes a challenging computational problem that hinders the aggregation of information about their bioactive moieties. We have developed an algorithm and software package called “Cheminformatics Tool for Probabilistic Identification of Carbohydrates” (CTPIC) that analyzes the covalent structure of a compound to yield a probabilistic measure for distinguishing saccharides and saccharide-derivatives from non-saccharides. CTPIC analysis of the RCSB Ligand Expo (database of small molecules found to bind proteins in the Protein Data Bank) led to a substantial increase in the number of ligands characterized as saccharides. CTPIC analysis of Protein Data Bank identified 7.7% of the proteins as saccharide-binding. CTPIC is freely available as a webservice at (http://ctpic.nmrfam.wisc.edu).

## Introduction

Changes in the composition or structure of saccharide compounds can alter their bioactivities^1–4^. Saccharide complexes, including glycans, have been identified as biomarkers of cancer^5–8^, Alzheimer^9,10^, and other conditions^11–13^. In addition, as we and other groups have shown, saccharide complexes can be used as reliable biomarkers of cardiometabolic diseases and systemic inflammation^14–18^. Global efforts have focused on organizing information about the bioactivities, structures, biosynthesis, and degradation patterns of saccharides and their conjugates in a variety of databases including Protein Data Bank (PDB)^19–21^, RCSB PDB Ligand Expo^22^, CCMRD^23^, and the KEGG glycan database^24^. One example of these efforts is the GlyGen Project (https://www.glygen.org) funded by the US National Institutes of Health as part of an international effort aimed at developing computational and informatics resources and tools for glycosciences research.

We have previously shown that assigning unique identifiers to chemical compounds is an essential step for aggregating information from different experimental and theoretical metabolomics databases^25,26^. Before developing such unique identifiers for saccharide complexes, a prerequisite step is to first identify whether a chemical compound has a saccharide origin. Distinguishing saccharide-derivatives from non-saccharide compounds is a challenging computational problem because saccharides complexes may undergo chemical reactions that result in major structural modifications^27^.

We present here an algorithm and software package called “Cheminformatics Tool for Probabilistic Identification of Carbohydrates” (CTPIC) that addresses the essential need for a method for identifying saccharides and their derivatives in a way that distinguishes them from compounds of non-saccharide origin. CTPIC provides two probabilistic scores to report similarities between a given chemical compound and saccharide structures: one score for the probability of the highest scoring fragment of the molecule, and another score for the entire molecule. Molecular fragments of a given compound are analyzed to identify fragments that resemble structures of saccharides. The number of atoms in the identified fragments over the total number of atoms in the compound are considered as the compound probability, which represents the fraction of the compound that is similar to saccharide structures. Among these fragments, the fragment that is most similar to saccharide structures is then used to calculate the fragment probability of the compound.

We demonstrate how this tool can be used to annotate the carbohydrate relatedness of compounds in a ligand structural library and to classify proteins as saccharide-binding on the basis of their structures.

## Results

### CTPIC: availability and use

The probabilistic algorithm has been developed in Python, and the source codes are publicly available through GitHub (https://github.com/htdashti/ctpic). In addition, the method is freely available through a web server (http://ctpic.nmrfam.wisc.edu) that accepts as its input the three-dimensional covalent structures of small molecules in SDF or MOL format^28^. After executing the probabilistic method in the background, the results are made available through the website. For each queried compound, the output report contains a list of molecular fragments that are found to be similar to known saccharides or their derivatives. The web server uses ALATIS^25,26^ unique atom identifiers in reporting these fragments. In addition, the web server utilizes the Open Babel^29^ package (http://openbabel.org) for identifying ligands in the RCSB PDB Ligand Expo^22^ library that are structurally similar to the queried compound. The result page on the website will report the top five most similar ligands and their corresponding protein-ligand complexes on the PDB website^19–21^.

### Validation of the approach for probabilistic identification of saccharide compounds

We show here that our method assigns high probabilities to known saccharides and low probabilities to non-saccharides. For a given structure file of a chemical compound, CTPIC identifies fragments of the compound that can be mapped to saccharide structures. The fragment with the highest probability of being a saccharide-derivative is called the best fragment, and its assigned probability is used to report the similarity score of the compound to saccharide structures. We used CTPIC to assess the probabilities for sets of known saccharide and non-saccharide compounds.

#### Analysis of known saccharide and non-saccharide compounds

**Fig. 1:**
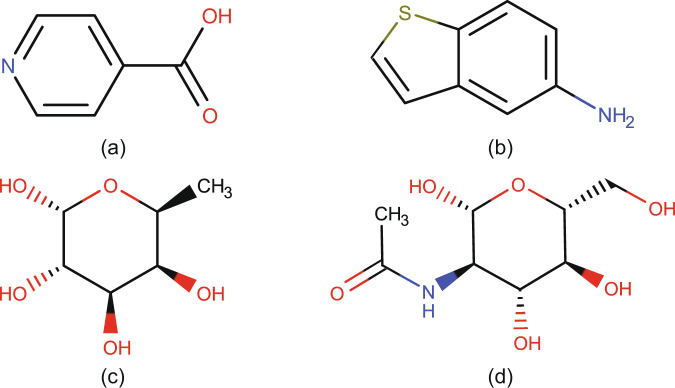
Examples of compounds analyzed by CTPIC. Non-saccharide compounds yielding scores of 0: (**a**) isonicotinic acid [C_6_H_5_NO_2_] and (**b**) 1-benzothiophen-5-amine [C_8_H_7_NS]. Saccharide compounds yielding scores of 1.0: (**c**) fucose [C_6_H_12_O_5_] and (**d**) N-acetylglucosamine [C_8_H_15_N_1_O_6_].

100 non-saccharide chemical compounds were extracted manually from the Maybridge Ro3 fragment library (https://www.maybridge.com/), and their 3D structures were obtained from the GISSMO website^25,30^. CTPIC assigned probabilities of zero to each of these compounds. Two examples of these compounds are shown in Fig. 1; results from the entire set of non-saccharide examples are on (http://ctpic.nmrfam.wisc.edu).

We selected 100 saccharide derivatives, including aldoses, ketoses, amino sugars, and intramolecular anhydrides, from an IUPAC publication on carbohydrate nomenclature^27^. CTPIC assigned high probabilities to these compounds (mean: 0.98, STD: 0.04). Two examples of these compounds are shown in Fig. 1; result from the entire set is available on the website.

These examples of non-saccharide and saccharide compounds show that the calculated probabilities can be used as an indicator of the similarity between given small molecules and saccharide structures. Therefore, the algorithm can be used as a binary classifier (saccharide vs. non-saccharide). On these examined sets of 100 saccharides and 100 non-saccharides, the accuracy of CTPIC, as a binary classifier, was 100%.

### Application of the approach to identifying saccharides in structural databases

#### Identification of compounds in the RCSB PDB Ligand Expo database that contain saccharide fragments

**Fig. 2:**
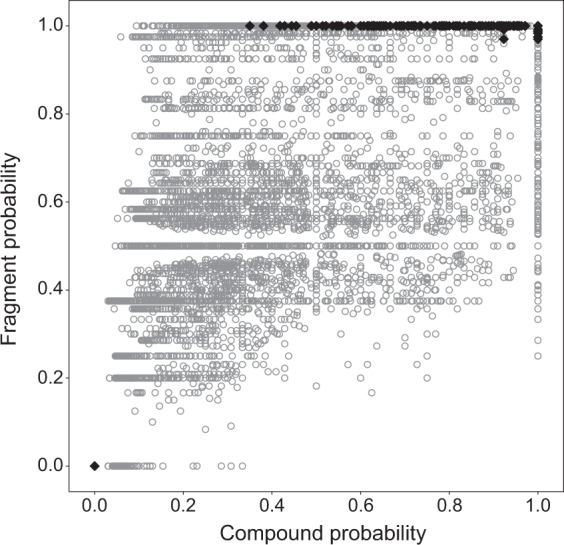
Scatter plot of calculated probabilities for the RCSB PDB Ligand Expo entries. The *y*-axis indicates the best fragment probability, and the *x*-axis shows the compound probability. In this plot, the 571 compounds that were annotated in this database as “saccharide” are shown as filled black diamonds, and the remaining compounds are shown as grey circles.

The RCSB PDB Ligand Expo^22^ is a database that contains three-dimensional structures of 29,993 small molecules (structure files downloaded on October 1, 2019) that have been found to be associated with structures of biological macromolecules deposited in the Protein Data Bank (PDB). 28,988 of these entries have been assigned to a “Component type” (Table 1). As indicated in the table, a total of 571 entries were annotated as “saccharide” (marked with asterisks: saccharide; D-saccharide; D-saccharide 1,4 and 1,4 linking; L-saccharide; L-saccharide 1,4 and 1,4 linking). We utilized CTPIC to analyze each of the 29,993 compounds in the RCSB PDB Ligand Expo database to determine their saccharide fragment and compound probability scores. These are shown as a scatter plot in Fig. 2. The complete list of the entries and their assigned probabilities are available on the website (http://ctpic.nmrfam.wisc.edu).

**Table 1:** Annotated components types archived in the RCSB PDB Ligand Expo.

Component Type	# entries	Component Type	# entries
non-polymer	26566	L-peptide linking	1182
* saccharide	200	D-peptide linking	123
* D-saccharide	299	peptide-like	539
* D-saccharide 1,4 and 1,4 linking	13	peptide linking	77
* L-saccharide	58	D-beta-peptide, C-gamma linking	1
* L-saccharide 1,4 and 1,4 linking	1	D-gamma-peptide, C-delta linking	1
RNA linking	287	L-gamma-peptide, C-delta linking	1
L-RNA linking	5	L-peptide COOH carboxy terminus	9
L-DNA linking	4	D-peptide NH3 amino terminus	2
DNA linking	405	L-beta-peptide, C-gamma linking	1
DNA OH 3 prime terminus	3	RNA OH 5 prime terminus	1
DNA OH 5 prime terminus	2	RNA OH 3 prime terminus	2
L-peptide NH3 amino terminus	13	NA	198

**Fig. 3:**
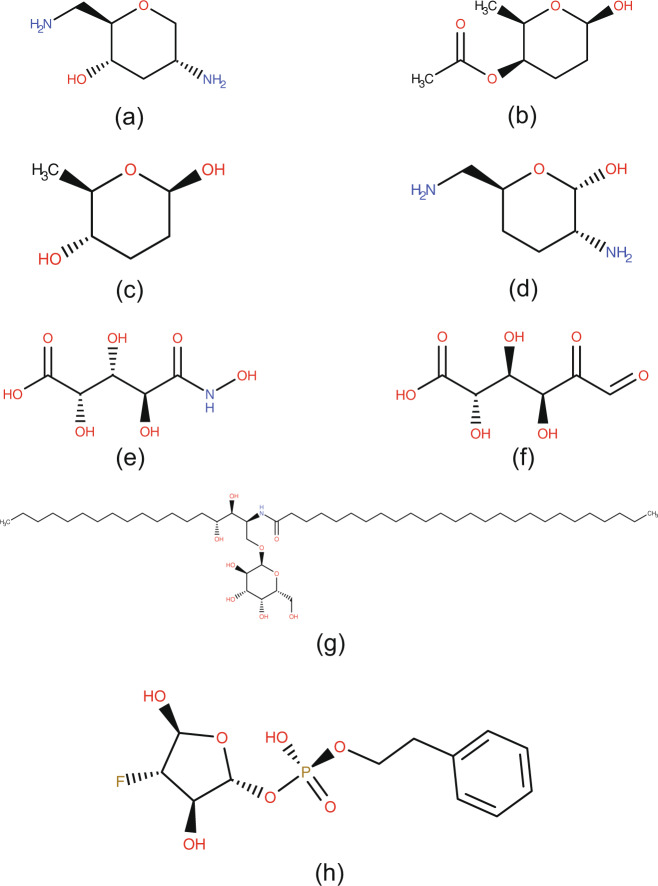
Examples of the saccharide compounds in RCSB PDB Ligand Expo database. (**a–d**) These compounds were assigned probabilities of “zero” due to the lack of sufficient oxygen atoms: (**a**) 2,6-diamino-2,3,6-trideoxy-α-D-ribo-hexopyranosyl, entry ID: *ADR*, formula: C_6_H_14_N_2_O_2_, (**b**) [O4]-acetoxy-2,3-dideoxyfucose, entry ID: *ARI*, formula: C_8_H_14_O_4_, (**c**) 2,3-dideoxyfucose, entry ID: *CDR*, formula: C_6_H_12_O_3_, (**d**) 3,4-dideoxy-2,6-amino-α-D galactopyranose, entry ID: *GE1*, formula: C_6_H_14_N_2_O_2_. **(e,f)** Compounds with fragment probabilities of 0.97: (**e**) D-arabinohydroxamic acid, entry ID: *HDL*, formula: C_5_H_9_NO_7_, compound probability: 0.92, (f) D-fructuronic acid, entry ID: *FIX*, formula: C_6_H_8_O_7_, compound probability: 1.00. **(g,h)** Compounds with the lowest compound probabilities: (**g**) n-[(1 s,2r,3 s)-1-[(α-D-galactopyranosyloxy) methyl]-2,3-dihydroxy heptadecyl] hexacosanamide, entry ID: *AGH*, formula: C_50_H_99_NO_9_, compound probability: 0.35, (h) (2 R,3 R,4 S,5 S)-4-fluoro-3,5-dihydroxytetra hydrofuran-2-yl 2-phenylethyl hydrogen S-phosphate, entry ID: 46Z, formula: C_12_H_16_FO_7_P, compound probability: 0.38.

CTPIC assigned fragment and compound probabilities of “zero” to five of the entries annotated as “saccharide”. One of these entries, entry ID *GTE* with the chemical formula “OH”, was mistakenly annotated as a saccharide. The remaining four entries, shown in Fig. 3a–d, represent compounds that the probabilistic method failed to identify as saccharide derivatives owing to their lack of sufficient diagnostic oxygen atoms. Apart from these five entries, the lowest fragment probability of the 571 entries annotated as “saccharide” was 0.97. The two entries with probability of 0.97 are shown in Fig. 3e,f; their lower than 1.0 score can be attributed to the structural modifications of their saccharide moieties.

Several entries annotated as “saccharide” received fragment probability of “1” but low compound probabilities. These entries contain a saccharide fragment modified by atoms that do not constitute a saccharide structure. The compounds with the lowest compound probabilities (0.35 and 0.38) corresponded to entry ID *AGH* and ID *46Z*, respectively. As shown in Fig. 3g,h, both of these entries contain a saccharide fragment; however, the long methylene chains in entry ID *AGH* and the phenyl ring in entry ID *46Z* resulted in the low compound probabilities.

**Fig. 4:**
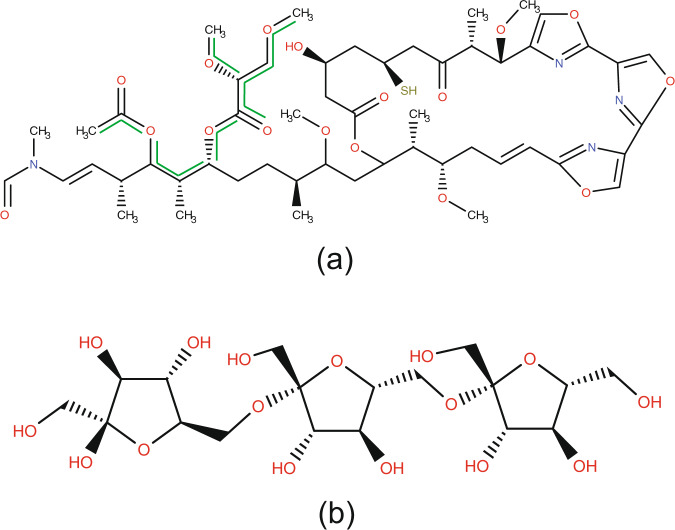
Two examples of entries from the RCSB PDB Ligand Expo that the probabilistic method suggests to annotate as saccharide-derivatives. (**a**) Mycalolide B, entry ID: *JQV*, formula: C_52_H_76_N_4_O_17_S, fragment probability: 0.97, compound probability: 0.35. A carbohydrate chain is indicated with green lines. (**b**) β-D-fructofuranosyl-(2->6)-beta-D-fructofuranosyl-(2->6)-beta-D-fructofuranose, entry ID: *0UB*, formula: C_18_H_32_O_16_ fragment and compound probabilities are equal to one.

Examination of these structures led us to choose fragment scores of 0.97 and higher, and compound scores of 0.35 and higher as the thresholds for designating a compound as having “saccharide” origin. According to this designation, the RCSB PDB Ligand Expo contains 4,553 compounds scored as “saccharide”, which is 3,982 more than the original number of 571. The entire set of compounds newly annotated as “saccharide” is available on the website. Compounds that exemplify the extremes of this classification range are shown in Fig. 4. Mycalolide B (entry ID *JQV*, Fig. 4a) received the lowest scores for “saccharide” designation. It contains a saccharide fragment (highlighted in green) plus extensive non-saccharide moieties. At the other end of the scale, β-D-fructofuranosyl-(2- > 6)-beta-D-fructofuranosyl-(2- > 6)-beta-D-fructofuranose (entry ID *0UB*, Fig. 4b) received fragment and compound probabilities of 1.0.

### Identifying saccharide binding proteins

Lectins and saccharide binding proteins are involved in many biological processes, including cell recognition, cell-cell adhesion, and immune functions^31–35^. In this section, we show another application of CTPIC for identification of these macromolecules by probabilistic annotation of small molecule with saccharide origin that bind to the proteins. To show how the method can be used for identifying saccharide binding proteins, we analyzed the cross references from the RCSB Ligand Expo to the PDB structural database of macromolecule complexes^19–21^. The majority of the small molecule structures stored in the Ligand Expo database are extracted from molecular complexes archived in the PDB, and the Ligand Expo database provides cross links between the small molecules and their corresponding macromolecule entries. Analyzing these cross references from the small molecules that are annotated by CTPIC as saccharides to the macromolecules provides a systematic path for identifying saccharide binding proteins in PDB. For example, the small molecule mycalolide B (Fig. 4a) is linked to the structure of rabbit actin protein (RCSB PDB entry ID *6MGO*, 10.2210/pdb6MGO/pdb). As indicated in the structure of the complex, the carbohydrate region highlighted in (Fig. 4a) binds to an active site of the protein at threonine-353 and methionine-357. We note that the research article of the RCSB PDB entry ID *6MGO*, with the structural resolution of 2.2 Å, has not been published yet, and therefore identifying this protein as a saccharide-binding protein was not possible through other means. RCSB PDB entry ID *0UB* (Fig. 4b) is linked to the RCSB PDB macromolecule entry ID *4FFI* (10.2210/pdb4FFI/pdb), which is reported in its associated research article as a saccharide binding proteins in plants^36^.

Because the probabilistic method can identify small molecules as saccharide-derivatives, the macromolecules that bind to these saccharides can be annotated as saccharide binding proteins or lectins. From the 4,553 annotated saccharides and saccharide-derivatives from the Ligand Expo database, 4,409 compounds were cross referenced to 12,297 unique RCSB PDB macromolecules (7.7% of the 158,998 entries archived in the database). The list of these saccharide-binding proteins is available on the website (http://ctpic.nmrfam.wisc.edu).

## Discussion

Because of the wide range of bioactivities of saccharides, compounds containing these moieties are at the center of numerous biochemical and biomedical investigations. Saccharide-containing molecules have been identified as biomarkers of disease and pathophysiological irregularities. Recent efforts from the glycomics community highlight the need for aggregating and compiling available metadata about these chemical compounds from across databases. We have introduced here a probabilistic method (CTPIC) for distinguishing compounds that contain saccharide moieties from those that do not. We have demonstrated the abillity of the probabilistic method to distinguish saccharides from non-saccharides and, more importantly, to identify saccharide fragments in chemical compounds that contain both saccharide-like and non-saccharide fragments. We have shown that CTPIC can be used to identify saccharide binding proteins on the basis of analysis of their binding ligands. This probabilistic method addresses an essential need for identifying saccharide complexes, and provides a platform for the design and development of unique identifiers for saccharides complexes and glycans.

## Methods

**Fig. 5:**
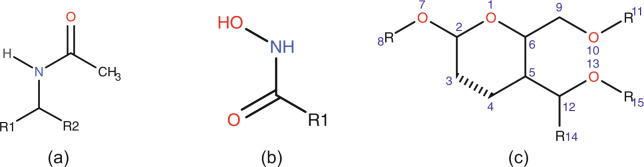
Examples of “saccharide fingerprints”. (**a**) Saccharide fingerprint for ring fragments. (**b**) Saccharide fingerprint for chain fragments. (**c**) Larger saccharide template. The dashed line bond between C3 and C4 in the ring indicates that the template can represent 5 or 6 membered rings. R8 can be a hydrogen or any other atom composite (e.g., CH_2_-, CH_3_). R11 and R15 can be any single or composite substructure. For R14 as a hydrogen, C9 and C12 would represent similar fingerprints, however, R14 can also be any heavy atom (e.g., O, OH, NH_3_).

The probabilistic software program (CTPIC) loads a three-dimensional structure file (in SDF or MOL format^28^) of the compound to be analyzed and uses the NetworkX library^37^ to convert the input structure file to a graph data structure, in which atoms are represented as nodes and edges of the graph represent covalent bonds between the atoms. The method looks for ring and chain molecular fragments in the given chemical compound and searches these fragments to identify substructures that we call “saccharide fingerprints”. We defined 37 molecular substructures, or saccharide fingerprints, that were extracted from an IUPAC carbohydrate nomenclature system^38^. Two examples of these saccharide fingerprints are shown in Fig. 5a,b; the complete list of the fingerprints used in the program is available on the website (http://ctpic.nmrfam.wisc.edu). Chain or ring molecular fragments that contain saccharide fingerprints are then called “saccharide templates”. Figure 5c shows an example of such templates: the 5- or 6-membered template ring is attached to three saccharide fingerprints (-OR, -CH_2_OR, -CHROR) with variable R-groups. The R-groups of the saccharide templates allow different atom compositions.

**Fig. 6:**
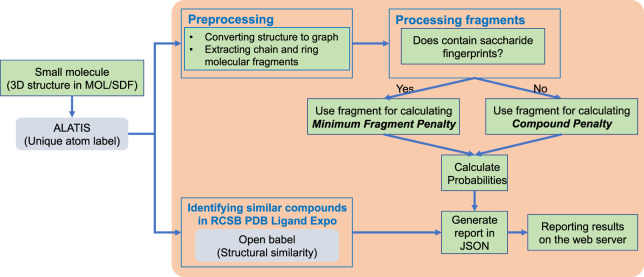
Workflow of the web server. For a given small molecule, the web server queries ALATIS to retrieve unique atom labels of the compound. The preprocessing module converts the structure file to a graph data structure, and extracts chain and ring molecular fragments. Then every fragment is analyzed to identify saccharide fingerprints. If no fingerprint found, the fragment is used in calculating a *compound penalty*. The molecular fragments that contain saccharide fingerprints are used in calculating the *minimum fragment penalty*. This penalty and the compound penalty are then used in calculating the probabilities. The web server reports the calculated probabilities and also lists every other calculated *fragment penalty* for the molecular fragments. In parallel, the web server uses the Open Babel package for identifying ligands with the highest structural similarities to the submitted molecule. These ligands from the RCSB PDB Ligand Expo are cross-referenced to the PDB molecular complexes. The outcome of this structural analysis reports proteins from PDB that bind to the identified ligands.

For a given compound, CTPIC calculates two probabilities: one that represents the fraction of the compound that can be mapped to saccharide templates (compound probability), and the other that represents the fractional similarity of molecular fragment of the compound with the most similar saccharide template (fragment probability). Figure 6 shows the overall workflow of the probabilistic method on the website. In this process, every chain or ring fragment of a given compound that contains one or more saccharide fingerprints is analyzed for its fragment probability. The fragments that do not contain any saccharide fingerprint serve to reduce the compound probability.

**Fig. 7:**
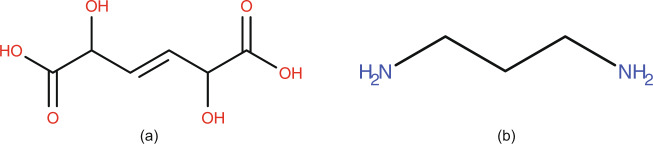
Structures of compounds used to illustrate the CTPIC algorithm. (**a**) (E)-2,5-Dihydroxyhex-3-enedioic acid, PubChem CID: 88515755, (**b**) 1,3-Diaminopropane, PubChem CID: 428.

After identifying saccharide fingerprints in a fragment and mapping the fragment to a saccharide template, the deviations of the fragment’s chemical formula from the aldehydes or ketones formula (C_n_[HOH]_m_) constitute fragment penalties. For example, (E)-2,5-dihydroxyhex-3-enedioic acid (Fig. 7a,formula: C_6_H_8_O_6_, PubChem CID: 88515755) is a chain compound, symmetric around a double bond and contains two carboxylic acids and two CHOH groups. These groups are saccharide fingerprints as defined in CTPIC, and, as such, the entire compound is considered as one molecular fragment mapped onto one saccharide template. The double bond is considered as a structural modification that resulted from the removal of two OH groups and counts as a penalty for the molecular fragment. In this example, the entire compound was mapped to one saccharide template; therefore, because there is no residual structure to be considered, the compound penalty is 0. These two types of penalties and the way they are used to calculate CTPIC probabilities are explained below.

### Calculating fragment penalty

When a ring or chain molecular fragment contains one or more saccharide fingerprints, the ratio of the number of required atom substitutions over the total number of atoms in the fragment is used as a penalty value. In this way, molecular fragments are assigned a penalty value that represents the lowest number of atom substitutions required to convert the fragment to a saccharide template. Of all molecular fragments that have been mapped to saccharide templates, the one with the minimum penalty is characterized by the *minimum fragment penalty*, i.e., a number between 0 and 1.

### Calculating compound penalty

The portion of an input molecule that cannot be mapped onto a saccharide template is used in calculating the “*compound penalty*”. The compound penalty indicates the ratio of the number of atoms in the input compound that could not be mapped to a saccharide template over the total number of atoms in the molecule. For example, 1,3-diaminopropane (Fig. 7b, chemical formula: C_3_H_10_N_2_, PubChem CID: 428) cannot be mapped to any saccharide fingerprint; and, therefore, the number of atoms that cannot be mapped to saccharide templates over the total number of atoms in the compound equals to 1, which is the compound penalty of this compound.

Because the *minimum fragment penalty* and the *compound penalty* are values between 0 and 1, we calculate the fragment and compound probabilities as one minus the penalties. Therefore, the fragment probability indicates the highest probability that a molecular fragment in the compound can be a saccharide-derivative, and the compound probability indicates the portion of the compound that can be mapped to saccharide templates.

## Data Availability

The output results on the RCSB PDB Ligand Expo are available on our website, and also have been deposited to the public domain through Open Science Framework [10.17605/OSF.IO/Y4U8M]^39^. The entries that were annotated as saccharides using the probabilistic method and their cross-references to the RCSB PDB macromolecule entries are also available on both our website and the Open Science Framework page^39^.

## Code availability

The cheminformatic tool for probabilistic identification of carbohydrate (CTPIC) program was developed using Python and is available on our website (http://ctpic.nmrfam.wisc.edu) as a web server. In addition, the source codes are available through GitHub (https://github.com/htdashti/ctpic).
